# A description of the structural determination procedures of a gap junction channel at 3.5 Å resolution

**DOI:** 10.1107/S0907444909014711

**Published:** 2009-07-10

**Authors:** Michihiro Suga, Shoji Maeda, So Nakagawa, Eiki Yamashita, Tomitake Tsukihara

**Affiliations:** a Institute for Protein Research, Osaka University, OLABB, 6-2-6 Furuedai, Suita, Osaka 565-0874, Japan; b Department of Life Science, University of Hyogo, Kamigohori, Akoh, Hyogo 678-1297, Japan

**Keywords:** gap junction channel, multi-crystal averaging, membrane proteins

## Abstract

Intercellular signalling is an essential characteristic of multicellular organisms. Gap junctions, which consist of arrays of intercellular channels, permit the exchange of ions and small molecules between adjacent cells. Here, the structural deter­mination of a gap junction channel composed of connexin 26 (Cx26) at 3.5 Å resolution is described. During each step of the purification process, the protein was examined using electron microscopy and/or dynamic light scattering. Dehydration of the crystals improved the resolution limits. Phase refinement using multi-crystal averaging in conjunction with noncrystallographic symmetry averaging based on strictly determined noncrystallographic symmetry operators resulted in an electron-density map for model building. The amino-acid sequence of a protomer structure consisting of the amino-terminal helix, four transmembrane helices and two extracellular loops was assigned to the electron-density map. The amino-acid assignment was confirmed using six selenomethionine (SeMet) sites in the difference Fourier map of the SeMet derivative and three intramolecular disulfide bonds in the anomalous difference Fourier map of the native crystal.

## Abstract 2

The structural determination procedures of a gap junction channel at 3.5 Å resolution are described, including the preparation of crystals, intensity data collection, data processing, phasing and structural refinement.

## 1. Introduction

Gap junctions, which are specialized regions in cellular membranes, contain hundreds of gap junction channels that allow small compounds such as ions, metabolites, nucleotides and small peptides to pass between cells (Kumar & Gilula, 1996 ▶). A gap junction channel spans two adjacent plasma membranes and is formed by end-to-end docking of two hemichannels, also referred to as connexons, each of which is composed of six connexin subunits (Harris, 2001 ▶). To date, more than 20 different genes encoding connexins have been identified in the human genome. Sequence-alignment analysis and a low-resolution structure obtained using electron microscopy suggest that the connexins have a four-transmembrane helical structure (Unger *et al.*, 1999 ▶; Fleishman *et al.*, 2004 ▶). Each gap junction channel exhibits a unique set of properties, including selectivity for small molecules, voltage-dependent gating and sensitivity to Ca^2+^, pH and phosphorylation (Simon & Goodenough, 1998 ▶; Saez *et al.*, 2003 ▶).

Despite numerous studies using two-dimensional electron microscopy to examine gap junction channels (Unger *et al.*, 1999 ▶; Fleishman *et al.*, 2004 ▶; Oshima *et al.*, 2007 ▶), the molecular mechanisms governing the functions of the connexins largely remain unknown and the structure of the connexins has yet to be determined at near-atomic resolution. Thus, a three-dimensional structure determined at high resolution is desirable.

It is often challenging to obtain crystals of membrane proteins that diffract to a high enough resolution to allow structural determination using the X-ray diffraction method. By optimizing the conditions for purification, crystallization and X-ray experiments, we obtained diffraction data of the human connexin 26 (Cx26) gap junction channel to 3.5 Å resolution. However, the crystal structure of human Cx26 gap junction channel could not be straightforwardly determined using routine procedures.

In this paper, we describe the procedures used to determine the three-dimensional structure of the human Cx26 gap junction channel, including sample preparation, crystal dehydration, intensity data collection and processing. In the structural determination, initial phase determination was performed using heavy-atom isomorphous replacement (IR) combined with anomalous dispersion, the locations of the methionine (Met) residues were determined using the difference Fourier method with a selenomethionine (SeMet) derivative crystal, phase extension was performed using noncrystallographic symmetry averaging (NCSA) in conjugation with multi-crystal averaging, structural refinement and successive Fourier synthesis were used to build the structural model and the disulfide bonds were located using the native anomalous dispersion method. Structural details and functional mechanisms have been described in a separate paper (Maeda *et al.*, 2009 ▶).

## 2. Methods

### 2.1. Preparation of crystals of the Cx26 gap junction channel

Human Cx26 was expressed without any tags using a con­ventional baculovirus/Sf9 expression system. The membrane fraction containing the gap junction was isolated from the collected cells in alkaline buffer containing 20 m*M* NaOH. The gap junction channel was isolated from the membrane fraction using detergent. Isolation, purification and crystallization of the Cx26 gap junction channel were performed as described elsewhere (Maeda *et al.*, 2009 ▶).

To improve the resolution limit, the crystals were dehydrated with increasing concentrations of triethylene glycol (TEG). The crystals were transferred into 100 µl buffer solution [16–18% polyethylene glycol (PEG) 200, 300 m*M* KCl, 0.1 *M* potassium phosphate (KP_i_), 10 m*M* dithiothreitol (DTT) and 0.2% undecyl-maltoside (UDM) at pH 7.5] and half of the buffer volume was replaced with buffer containing an additional 2% TEG. This procedure was repeated every 15–30 min until the final concentration of TEG reached 25–30%. After the crystals had been equilibrated in the final buffer for 1 or 2 d, they were frozen in liquid nitrogen.

### 2.2. Intensity data collection and processing

Most of the data sets were collected using a DIP 2040 imaging-plate detector (Bruker AXS) on the BL44XU beamline at SPring-8. The data sets collected at SPring-8 were processed and scaled using *DENZO* and *SCALEPACK* (Otwinowski & Minor, 1997 ▶) and *SCALA* from the *CCP*4 package (Collaborative Computational Project 4, Number 4, 1994 ▶). A native data set from ten isomorphous crystals equili­brated in crystallization solution containing 30% TEG was collected at 3.5 Å resolution (native I). Another native crystal was prepared after equilibrating in 25% TEG for phase refinement *via* multi-crystal averaging. The diffraction data for this crystal were collected at 4.0 Å resolution (native II). To detect anomalous dispersion effects from S atoms in the native crystal, another native data set was acquired at 4.0 Å resolution using a Pilatus 6M detector on the X06SA beamline at the Swiss Light Source (SLS), Paul Scherrer Institute, Villigen, Switzerland using 1.7000 Å X-rays. The data set collected at SLS was integrated and scaled with *XDS* and *XSCALE* (Kabsch, 1993 ▶). Derivative crystals that were isomorphous to each native crystal were prepared by soaking the native crystals in a cryoprotectant solution containing the same TEG concentration as that for the native crystal. Three data sets for a tantalum derivative (Ta_6_Br_14_ derivative for multiple-wavelength anomalous dispersion) equilibrated with 30% TEG were acquired by tuning the X-rays to 0.9000 Å (remote), 1.2526 Å (peak) and 1.2552 Å (edge). SeMet-derivative crystals were soaked in crystallization solution containing 30% TEG and diffraction data sets were collected using X-rays at 0.9000 Å (remote) and 0.9790 Å (edge).

### 2.3. Phase-determination and structural refinement procedures

The self-rotation function was calculated at 6 Å resolution to determine the orientation of the noncrystallographic sixfold axis using *POLARRFN* from the *CCP*4 program suite. The initial phases of the native I crystal were determined using the single isomorphous heavy-atom replacement (SIR) method coupled with the anomalous dispersion (SIRAS) method. Assuming the Ta cluster to be a single atom, the positional parameters and *B* factor of the Ta cluster were refined using *SHARP* (Bricogne *et al.*, 2003 ▶). The phases were refined and extended from 15 to 3.5 Å resolution by NCSA, solvent flattening and histogram matching using *DM* from the *CCP*4 program suite. For the first phase refinement, the noncrystallographic symmetry (NCS) parameters of the sixfold axis were fixed at (ω, ϕ) = (30.3°, 180.0°).

To determine the direction of the sixfold NCS axis accurately, the preliminary refined phases were extended from 5.0 to 3.5 Å resolution by NCSA for each ω value from 28.0° to 34.0° in steps of 0.1° (ϕ = 180.0°, κ = 60.0°). Progress in the phase refinement was inspected by calculating the *R* factor and the correlation coefficient: *R* = 
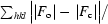
 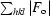 and CC = 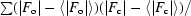 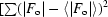 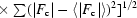. When selecting the best ω angle for the sixfold axis, the second phase refinement was carried out from 15 to 3.5 Å using NCSA.

The refined phase set at 3.5 Å resolution was used to calculate the difference Fourier map of the SeMet derivative with coefficients [*F*_o_(remote) − *F*_o_(edge)]exp(*i*α_c_), where α_c_ is the refined phase. 36 SeMet sites were identified in the difference Fourier map. 30 sites were included in the phase calculation, whereas the other six sites were used to monitor the phase improvement by estimating their electron densities in the difference Fourier map.

Further phase refinement was performed with multi-crystal averaging and sixfold NCSA coupled with solvent flattening and histogram matching using *DMMULTI* from the *CCP*4 program suite. Two native crystals, a tantalum-derivative crystal and an SeMet-derivative crystal were used in the multi-crystal averaging. The initial phases for the phase refinement were determined at 6.0 or 7.0 Å resolution, those of the native I crystal were determined using the SIRAS method, those of the native II crystal were determined using the SIR method and those of the tantalum-derivative and SeMet-derivative crystals were determined using the MAD method. The phases were extended from 6.0 to 3.5 Å resolution and an electron-density map at 3.5 Å resolution was calculated using the observed structure factors of native I and the refined phases.

A structural model of Cx26 was built into the electron-density map calculated at 3.5 Å resolution using *O* (Jones *et al.*, 1991 ▶) and *Coot* (Emsley & Cowtan, 2004 ▶). Each amino acid was assigned to the electron-density map based on the specific features of the bulky amino-acid residues and the locations of the SeMet residues. Structural refinement was carried out under a noncrystallographic sixfold symmetry restraint using the *Crystallography & NMR System* (Brünger *et al.*, 1998 ▶) and *REFMAC* (Murshudov *et al.*, 1997 ▶). Prior to calculation of the native anomalous difference map, each cysteine and methionine residue in the refined structure was replaced with an alanine residue and the new structure was refined with the S anomalous data set acquired with 0.9850 Å X-rays in order to reduce the contribution of the S atoms from the Cys residues to the phases of the native crystal. An anomalous difference Fourier map was calculated with co­efficients (|*F* ^+^| − |*F* ^−^|)exp[*i*(α − π/2)] to confirm the sites of the disulfide bonds in the extracellular region of the protein; |*F* ^+^| and |*F* ^−^| are the observed structure factors of (*h k l*) and (−*h* −*k* −*l*) for the native crystal using 1.7000 Å X-rays, while α is the refined phase of the structure containing the substituted Ala residues.

All figures were generated using *PyMOL* (DeLano, 2002 ▶).

## 3. Results and discussion

### 3.1. Preparation of crystals and data collection

**Figure 1:**
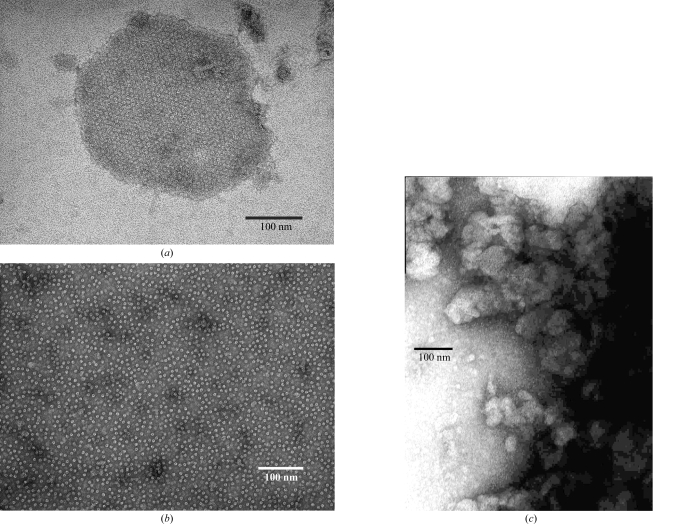
Electron-microscopic images of the gap junction channels. (*a*) An electron-microscopic image of the membrane fraction obtained following alkaline treatment of the cells. (*b*) An electron-microscopic image of the gap junction channel solubilized with DDM. (*c*) An electron-microscopic image of the gap junction channel solubilized with octyl-β-d-glucoside. The samples were aggregated.

Fig. 1 ▶(*a*) shows an electron micrograph of the membrane fraction after alkaline treatment of the cells. A plaque structure exhibiting a two-dimensional array of gap junction channels is clearly visible. The membrane fraction was solubilized with various detergents in solubilization buffer. In order to select a suitable detergent, gap junction channels solubilized under different conditions were inspected using the electron microscope. Figs. 1 ▶(*b*) and 1 ▶(*c*) are typical electron micrographs. Of the 44 detergents tested, dodecyl-maltoside (DDM), UDM, sucrose monododecanoate, CYMAL-5, CYMAL-6 and CYMAL-7 preserved the structural features of the gap junction channels that had been observed in electron micrographs. Because dynamic light-scattering (DLS) analysis indicated that the samples treated with UDM and DDM were monodisperse, these detergents were selected for solubilization of Cx26 from the membrane fraction. The purified Cx26 structures were uniform in size and shape (Fig. 1 ▶ *b*). We also expressed and purified hexa-His-tagged Cx26, but found that Cx26 often precipitated in the presence of nickel ion. Thus, we did not use the hexa-His tag to prepare the Cx26 sample.

The dehydration process shrunk the crystals by 13% in unit-cell volume, from unit-cell parameters *a* = 179, *b* = 117, *c* = 157 Å, β = 112° to *a* = 167, *b* = 112, *c* = 155 Å, β = 114°, and markedly improved the resolution limit of the crystals from 7 Å resolution to 3.5 Å resolution. Although annealing (Harp *et al.*, 1998 ▶; Yeh & Hol, 1998 ▶) or additive screening slightly improved the resolution limit of the crystals, we did not obtain any crystals that diffracted to a resolution better than 6.0 Å. The dehydration procedure was key to obtaining the intensity data that were available for the structural determination.

The isomorphism of the Cx26 crystals was highly dependent on the concentration of TEG and longer equilibration periods in crystallization buffer containing the final concentration of TEG improved the isomorphism of the crystals. Many iso­morphous crystals were required in order to acquire higher resolution data by a long exposure period for each spot. The intensity data set of native I were acquired using ten crystals, an X-ray exposure time of 30 s and an oscillation angle of 1.0° on BL44XU of SPring-8. The accuracy of the intensity data in the high-resolution range was markedly improved by the longer exposure time. Despite the poor quality of the crystals, 3.5 Å resolution data were successfully obtained with an *R*_merge_ value of 37.8%, a completeness of 94.4% and an averaged redundancy of 5.9 in the highest 3.63–3.50 Å resolution shell. The collected intensity data for native and derivative crystals are summarized in Table 1 ▶.

### 3.2. Structure determination

#### 3.2.1. Crystal packing, orientation of the noncrystallographic sixfold axis and location of the Met residues

**Figure 2:**
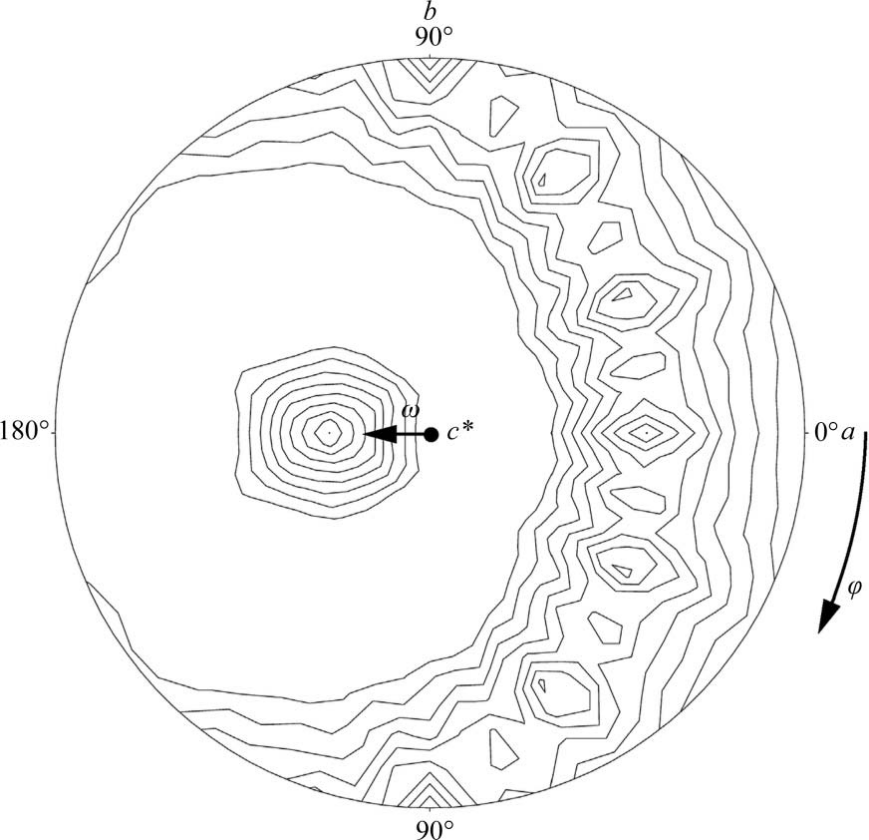
Stereo diagram of the self-rotation function for twofold rotational symmetry (κ = 180° section) calculated at 6.0 Å resolution. The maximum value was normalized to 100 and contours were drawn at intervals of 10 starting from 10.

The native I crystal belonged to the monoclinic space group *C*2, with unit-cell parameters *a* = 167.6, *b* = 111.2, *c* = 155.4 Å, β = 114.0°. Assuming that the unit cell contains two gap junction channels each consisting of 12 connexins, *V*_M_ (Matthews, 1968 ▶) was 4.4 Å^3^ Da^−1^, which is reasonable for membrane proteins (Tomizaki *et al.*, 1999 ▶). The native Patterson function calculated at 6 Å resolution showed no prominent peaks except at the origin and (1/2, 1/2, 0), which corresponded to the face-centred symmetry operation. The self-rotation function at (ω, ϕ) = (30.3, 180.0°) exhibited peaks at κ = 60.0, 120.0 and 180.0°, which indicated a noncrystallographic sixfold axis on the *ac* plane leaning toward the *c* axis by approximately 30° from the c* axis. A section of the self-rotation function at κ = 180.0° is depicted in Fig. 2 ▶. The self-rotation function produced peaks at (ω, ϕ, κ) = (30.0, 180.0, 180.0°), (90.0, 90.0, 180.0°), (75.4, 63.5, 180.0°), (64.4, 33.7, 180.0°), (60.0, 0.0, 180.0°), (64.4, 326.3, 180.0°) and (75.4, 296.5, 180.0°). The first peak was included in the sixfold axis. The second peak was a crystallographic twofold axis along the *b* axis. The other peaks were related to the crystallographic twofold axis by the sixfold axis at (ω, ϕ) = (30.3, 180.0°). The Patterson function and the self-rotation function indicated that a gap junction channel with a point group of sixfold dihedral symmetry (*D*6) was at the origin and its twofold axis coincided with the crystallographic twofold axes.

The *R*_iso_ values [*R*_iso_ = 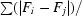 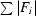] from native I to the derivative of native I, the SeMet derivative (remote) and the Ta_6_Br_14_ derivative for MAD (remote) were 0.073, 0.221 and 0.147, respectively, at 6.0 Å resolution. The SeMet derivative and Ta_6_Br_14_ derivative for MAD exhibited lower iso­morphism against native I than the Ta_6_Br_14_ derivative of native I. Therefore, the phases of native I calculated by the multiple isomorphous replacement with anomalous scattering (MIRAS) method using these derivatives at 6.0 Å resolution were not improved compared with those calculated by the SIRAS method with the derivative of native I. Consequently, we applied the SIR method followed by NCSA for phasing.

**Figure 3:**
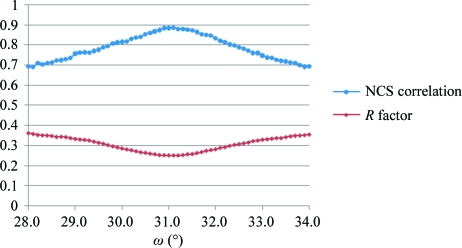
Plots of *R* factor and correlation coefficients *versus* ω of NCSA phase extension. The NCSA phase extension was performed from 5 to 3.5 Å at each ω of the sixfold axis. The phase extension was carried out in 100 steps and NCSA was iterated by 20 cycles at each resolution. The initial phases for each NCSA were the phases obtained by the phase extension of the SIRAS phases from 15 Å resolution. The best *R* factor and correlation coefficient were obtained at ω = 31.1°.

In the first phase extension from 15 to 3.5 Å resolution, the sixfold NCS parameters were fixed at (ω, ϕ) = (30.3, 180.0°) as obtained from the rotation function. The conventional *R* and the CC at 3.5 Å resolution were 0.289 and 0.848, respectively. Since the electron-density map composed at 3.5 Å resolution was too vague to build a model, an accurate orientation of the sixfold NCS axis was determined. Fig. 3 ▶ shows the plots of *R* and CC calculated by the NCSA phase extension from 5.0 to 3.5 Å resolution with each value of ω of the sixfold axis. The plots of *R* and CC *versus* ω showed that the most reliable ω value was 31.1°. The second phase extension was carried out to 3.5 Å resolution starting from 15 Å resolution with the strictly determined sixfold NCS parameters of ω = 31.1° and ϕ = 180.0°, resulting in an improvement of *R* to 0.262 and of CC to 0.891.

**Figure 4:**
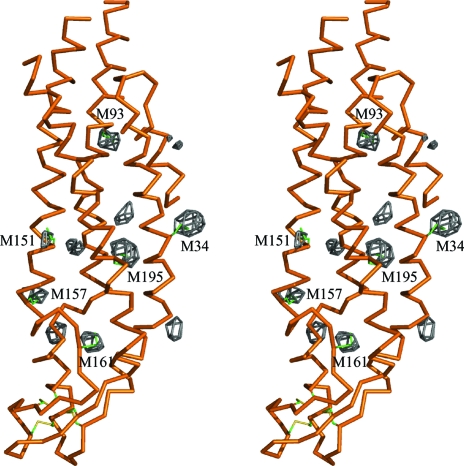
Stereoscopic drawing of the difference Fourier map of the SeMet derivative calculated at 4.0 Å resolution. The map is contoured at 4.0σ. All of the selenium sites, except for the N-terminal SeMet, identified in high-electron-density peaks were associated with a residue number. Peaks without a residue number result from Se atoms in other protomers.

In the difference Fourier map of the SeMet derivative, 36 of 42 Met sites in the protein molecule were identified as selenium peaks higher than 4σ of the electron-density distribution (Fig. 4 ▶). The six N-terminal Met1 sites could not be located in the map, probably because of their disordered structures. The six Met34 sites were excluded from further phase-determination steps and were instead used to monitor the phase improvement by their peak heights in the electron-density maps.

#### 3.2.2. Further phase refinement using multi-crystal averaging

**Figure 5:**
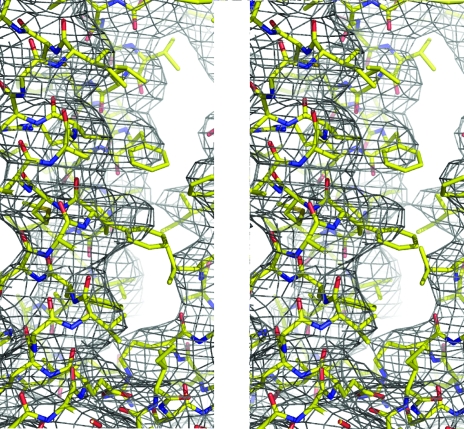
DM map calculated with the phases refined by multi-crystal averaging using *DMMULTI*. The electron-density map was calculated at 3.5 Å resolution and contoured at 0.7σ.

After repeating the multi-crystal averaging, the averaged peak height for the six SeMet sites in the difference Fourier map of the SeMet derivative which was calculated with the refined phases was 6.1σ of the electron-density distribution, whereas the peak of the phases refined with the single native data set was 4.5σ. In the Fourier electron-density map, aromatic residues showed bulky electron-density con­tours (Fig. 5 ▶). The electron-density map calculated from the refined phases by multi-crystal averaging using *DMMULTI* was significantly improved in comparison with that calculated using a single native data set in which the phases were refined using *DM*. Multi-crystal averaging among crystals belonging to the same space group was successfully applied to the phase-refinement procedure, as reported by Lescar *et al.* (2001 ▶) and Jeruzalmi *et al.* (2001 ▶), and it was a key step in obtaining sufficient electron density to enable us to build an atomic model.

**Figure 6:**
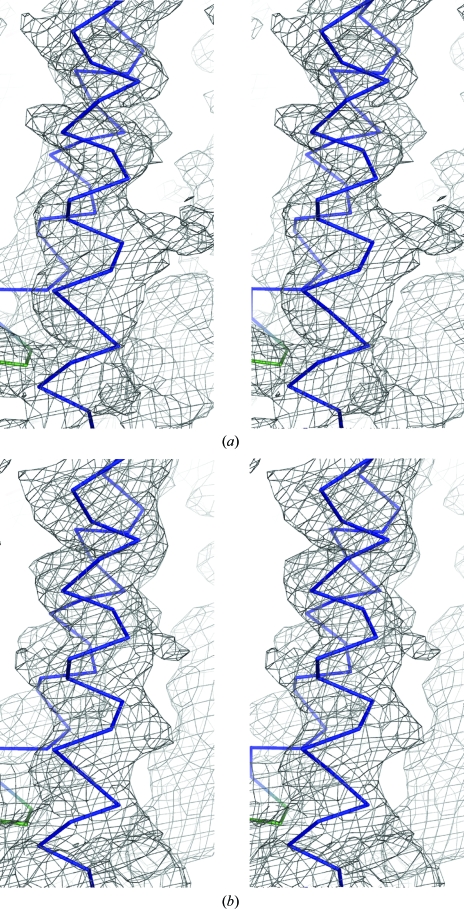
DM maps of the same region of the structure calculated with (*a*) refined phases from a single data set for native I using *DM* and (*b*) refined phases from two data sets from native I and native II using *DMMULTI*. Both maps are calculated at 3.5 Å resolution and contoured at 1.5σ. The latter showed an improved electron-density distribution for the helical structure.

To inspect the efficiency of the multi-crystal averaging for phase refinement, three types of test calculations were performed. Prior to the test calculation, the phases of native I, native II, SeMet and the Ta_6_Br_14_ derivative for MAD were prepared at 15 Å resolution. In the first case, the initial phases of a native data set, native I, were refined and extended from 15 to 6.0 Å by 200 steps of NCSA using *DM*. In the second case, the initial phases of two data sets, native I and a derivative, were combined using *SIGMAA* to generate phases of native I at 15 Å resolution and the phases of the native data were refined and extended to 6 Å resolution by 200 steps of NCSA using *DM*. In the third case, the initial phases of two data sets, native I and native II or a derivative, were refined together and extended to 6 Å resolution by 200 steps of multi-crystal averaging using *DMMULTI*. The quality of the refined phases was judged from the validity of the electron-density map and from the averaged peak heights of the six SeMet34 sites in the difference Fourier map of the SeMet derivative (Figs. 6 ▶ *a* and 6 ▶ *b* and Table 2 ▶). Judging by the peak heights of the selenium sites (Table 2 ▶), the phases refined using *DMMULTI* in the third case were significantly better than those refined using *DM*. The electron-density map of Fig. 6 ▶(*b*) calculated using *DMMULTI* exhibits more sound helical features than that of Fig. 6 ▶(*a*) calculated using *DM*. These results suggest that even if crystals are in the same space group, multi-crystal averaging using *DMMULTI* is available for phase refinement.

Although the *R*_iso_ value of 0.097 between native I and native II was smaller than those between native I and the other derivatives, inter-crystal averaging with native II effectively improved the electron-density distribution, as was obtained with the other derivatives. This suggests that a data set from non-isomorphous native crystals can be included efficiently in phase refinement by multi-crystal averaging in general. The availability of the non-isomorphous crystals, however, should be inspected by unbiased criteria against a model structure as is the present case with the peak heights for Met34. Protein crystals of membrane proteins tend to lose isomorphism when the preparation, crystallization and freezing conditions are changed slightly. Although this may be a difficult problem in general, it provides an opportunity for phase improvement using multi-crystal averaging and *DMMULTI*.

#### 3.2.3. Model building and location of the disulfide bonds

**Figure 7:**
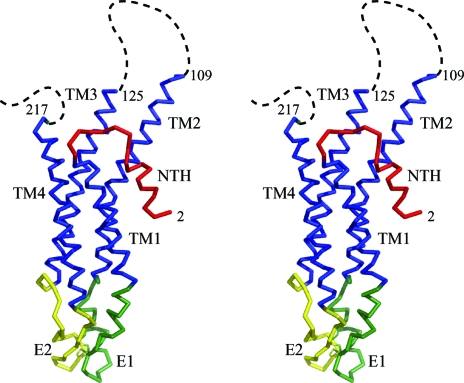
A stereoscopic C^α^ drawing of the Cx26 protomer. The short N-terminal helix (NTH) is shown in red, the transmembrane (TM) regions are shown in blue (TM1, TM2, TM3 and TM4) and the two extracellular loops are shown in green and yellow (E1 and E2). Dashed lines represent disordered regions.

The backbone of the protein was traced in the electron-density map calculated from the refined phases with multi-crystal averaging. The locations of the Met residues corresponded to the assigned SeMet sites in the difference Fourier map of the SeMet derivative and aromatic residues were located in the bulky electron-density cages of the electron-density map refined using *DMMULTI*. The native anomalous difference Fourier map successfully located three disulfide bonds, each bridging between two extracellular loops (E1 and E2). Con­sequently, the chain traces of E1 and E2 were confirmed by the native anomalous difference Fourier map. The assignment of the four transmembrane helices in one protomer differed from the previous model (Fleishman *et al.*, 2004 ▶). Successive Fourier transformations resulted in a short helix in the N-terminal region. The connecting loop between the short N-terminal helix and transmembrane helix 1 was very flexible in a low-electron-density region, which agreed with the NMR solution structure of the Cx26 N-terminal peptide (Purnick *et al.*, 2000 ▶). Of the 226 residues in each molecule, the atomic parameters of residues 2–109 and 125–217 converged well during the refinement (Fig. 7 ▶ and 8 ▶). Residues 110–124 and 218–226 could not be located because of poor electron density. Sixfold NCS restraints in the structural refinement affected the *R*_free_ value, which was close to the *R* value. The final refinement statistics are summarized in Table 1 ▶.

**Figure 9:**
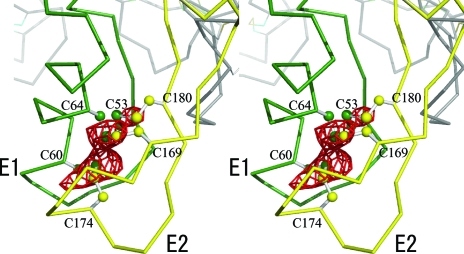
A stereoscopic drawing of the native anomalous difference map calculated at 4.0 Å resolution and drawn at 8.0σ. The peaks correspond to the three intraprotomer disulfide bonds that bridge the two extracellular loops; the two extracellular loops are shown in green and yellow (E1 and E2).

When the resolution of the structural analysis is as low as 3.5 Å, as is the case for the present crystal, amino-acid assignment should be confirmed by several different methods. Since almost all proteins contain Cys or Met, S anomalous difference Fourier synthesis can almost always be used to assign the amino-acid sequence. In the present study, we devised a way to locate S atoms in the anomalous difference Fourier map as follows. (i) Low-energy X-rays of 1.7 Å wavelength were used to increase the anomalous dispersion effect of S atoms. (ii) The intensity data were collected in the inverse-beam mode, which records Friedel pairs on two consecutive images to reduce the influence of radiation damage on the Bijvoet difference. (iii) To reduce systematic error in the Bijvoet difference derived from differences in experimental conditions, only reflections for which both Friedel pairs were recorded in inverse-beam mode were used. (iv) In order to make absorption effects less serious in phasing, a reference data set for accurate scaling and phase calculation was also collected from the same crystal using 0.9850 Å X-­rays. (v) The phases of the high-energy data set were calculated using the molecular-replacement method with the refined model in which each Cys and Met residue was replaced with an Ala residue and the phases of the high-energy data were used for the anomalous difference Fourier map of low-energy data in order to reduce the effect of the phasing error. (vi) The anomalous difference map calculated at 4.0 Å was averaged using *AVE* (Kleywegt *et al.*, 2001*a* ▶,*b* ▶) based on analysis of NCS operators determined at 3.5 Å. Consequently, the top three peaks for each protomer were 10.2σ, 9.8σ and 8.7σ of the electron-density distribution, which agreed with the positions of the disulfide bonds in the model; no com­parable peaks were detected. The locations of the three intramolecular disulfide bonds in the extracellular domains (Foote *et al.*, 1998 ▶) were confirmed in the map. Six relatively high peaks of 7.5σ, 6.5σ, 6.3σ, 6.1σ, 5.8σ and 4.5σ were observed for each protomer; these corresponded to the six Met sites identified in the SeMet derivative (Fig. 9 ▶). The present success in the assignment of S atoms implies that the native S anomalous difference Fourier method is suitable for determining the location of Met and Cys residues, even in a poorly diffracting crystal, under the conditions used for the X-­ray experiment and structural analysis in the present study.

## Supplementary Material

PDB reference: gap junction channel, 2zw3, r2zw3sf

## Figures and Tables

**Figure 8:**
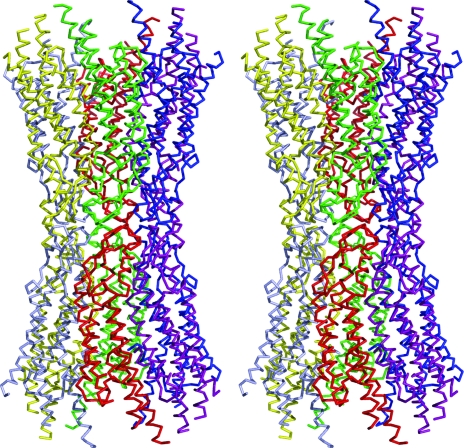
A stereoscopic C^α^ drawing of the Cx26 gap junction channel. The two hemichannels are related by a crystallographic twofold axis along the horizontal axis. The two protomers related by the twofold axis are depicted in the same colour.

**Table 1:** Statistics for the crystallographic data collection and refinement of the Cx26 gap junction channel Values in parentheses are for the last shell.

Crystal	Native I	Ta_6_Br_14_ derivative of native I	Native II	Ta_6_Br_14_ derivative of native II	SeMet derivative (edge)	SeMet derivative (remote)	Ta_6_Br_14_ derivative for MAD (peak)	Ta_6_Br_14_ derivative for MAD (edge)	Ta_6_Br_14_ derivative for MAD (remote)	S anomalous (high energy)	S anomalous (low energy)
Initial phase for multi-crystal averaging (phasing)	Native I (SIRAS)		Native II (SIR)		SeMet derivative (MAD)		Ta_6_Br_14_ derivative for MAD (MAD)				
Data-collection statistics											
No. of crystals	10	2	2	4	1†	1†	2	1†	1†	1	2
Space group	*C*2	*C*2	*C*2	*C*2	*C*2	*C*2	*C*2	*C*2	*C*2	*C*2	*C*2
Unit-cell parameters											
*a* (Å)	167.6	167.3	170.7	168.7	170.7	171.0	169.0	168.7	170.7	166.7	166.7
*b* (Å)	111.2	110.6	111.6	111.6	115.5	115.0	110.9	111.6	111.6	110.1	110.1
*c* (Å)	155.4	154.2	157.2	156.2	157.6	157.5	156.5	156.2	157.2	155.7	155.7
β (°)	114.0	114.3	114.0	113.4	113.7	113.8	114.4	113.4	114.0	114.4	114.4
s.d./unit-cell parameter‡ (%)	0.56			0.36							
Wavelength (Å)	0.9000	1.2526	0.9000	0.9000	0.9790	0.9000	1.2526	0.9000	0.9000	0.9850	1.7000
Resolution (Å)	200–3.50 (3.63–3.50)	200–6.00 (6.21–6.00)	200–4.00 (4.14–4.00)	200–7.00 (4.14–4.00)	200–4.00 (4.14–4.00)	200–4.00 (4.14–4.00)	200–4.00 (4.14–4.00)	200–4.00 (4.14–4.00)	200–4.00 (4.14–4.00)	200–3.50 (4.00–3.50)	200–4.00 (4.50–4.00)
Observed reflections	348035 (18261)	41085 (3433)	92816 (6526)	33075 (3326)	63815 (5358)	61306 (4766)	139511 (8088)	60030 (4130)	64219 (5005)	109122 (36329)	565283 (162661)
Independent reflections	31138 (3095)	6372 (613)	22327 (2105)	3964 (396)	21392 (2061)	21088 (1986)	21177 (1881)	20602 (1796)	21088 (2002)	31778 (10407)	42471 (12574)
*I*/σ(*I*)	26.1 (3.1)	35.4 (6.2)	15.3 (1.0)	38.8 (14.2)	13.1 (1.3)	12.9 (1.3)	20.2 (0.74)	14.0 (0.65)	9.3 (0.59)	7.51 (0.56)	13.53 (1.85)
Averaged redundancy	11.2 (5.9)	6.4 (4.5)	4.2 (3.1)	8.4 (8.4)	3.0 (2.6)	2.9 (2.4)	6.6 (4.3)	2.9 (2.3)	3.0 (2.5)	3.4 (3.4)	13.3 (12.9)
Completeness (%)	94.1 (94.9)	97.1 (94.5)	90.3 (95.1)	91.6 (93.2)	93.1 (90.4)	91.3 (86.4)	96.7 (87.6)	99.1 (98.4)	96.4 (92.3)	97.4 (97.1)	99.6 (99.2)
*R*_merge_§ (%)	8.5 (37.8)	5.2 (16.8)	9.4 (70.8)	5.9 (13.1)	8.6 (68.5)	7.0 (58.3)	9.4 (>100)	9.2 (93.2)	11.0 (>100)	10.6 (>100)	14.4 (>100)
Refinement statistics											
*R*_work_¶/*R*_free_	0.337/0.351										
No. of atoms	29595										
*B* factors (Å^2^)	137.2										
R.m.s. deviations											
Bond length (Å)	0.012										
Bond angle (°)	1.352										
Ramachandran plot (%)											
Favoured region	82.1										
Allowed region	16.8										
Generously allowed region	1.0										
Disallowed region	0.0										

† Edge data and remote data were collected from the same crystal.

‡ The average of standard deviation/unit-cell parameter was calculated.

§ *R*_merge_ = 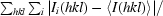 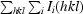, where *I*_*i*_(*hkl*) is the intensity of the reflection and 〈*I*(*hkl*)〉 is the mean intensity of a group of equivalent reflections.

¶ *R*_work_ = 
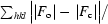
 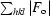, where *F*_o_ and *F*_c_ are the measured and calculated structure factors, respectively.

**Table 2:** Peak heights at the Met34 sites from the six protomers and their averaged values in various difference Fourier maps calculated at 6.0 Å resolution Data set I is the data set whose phases were given as initial phases. Data set II is the data set whose phases were refined. Data sets i, ii, iii and iv correspond to native I, native II, SeMet and Ta_6_Br_14_ derivative for MAD, respectively.

Data set		Peak height						
I	II	A	B	C	D	E	F	Average
*DM*								
i	i	5.2	4.5	4.7	3.8	4.2	4.8	4.5
i, ii	i	5.6	5.1	3.7	4.6	4.0	4.8	4.6
i, iii	i	6.1	5.7	4.4	3.7	5.5	4.9	5.1
i, iv	i	5.2	5.2	3.5	3.1	5.3	4.3	4.4
*DMMULTI*								
i, ii	i, ii	6.5	5.9	5.5	5.2	4.9	6.7	5.8
i, iii	i, iii	7.4	5.8	5.6	4.5	5.6	7.3	6.0
i, iv	i, iv	6.5	6.6	4.7	5.1	5.2	5.5	5.6
